# Roadmap of electrons from donor side to the reaction center of photosynthetic purple bacteria with mutated cytochromes

**DOI:** 10.1007/s11120-023-01059-1

**Published:** 2023-11-30

**Authors:** M. Kis, T. Szabó, J. Tandori, P. Maróti

**Affiliations:** 1 Balaton Limnological Research Institute, Klebelsberg K. Utca 3, Tihany, 8237 Hungary; 2 Institute of Medical Physics, University of Szeged, Korányi Fasor 9, Szeged, 6720 Hungary

**Keywords:** Bacterial photosynthesis, Photoreactions, Cyclic electron transfer, Fluorescence induction, Relaxation of fluorescence

## Abstract

In photosynthetic bacteria, the absorbed light drives the canonical cyclic electron transfer between the reaction center and the cytochrome *bc*_1_ complexes via the pools of mobile electron carriers. If kinetic or structural barriers hinder the participation of the *bc*_1_ complex in the cyclic flow of electrons, then the pools of mobile redox agents must supply the electrons for the multiple turnovers of the reaction center. These conditions were achieved by continuous high light excitation of intact cells of bacterial strains *Rba. sphaeroides* and *Rvx. gelatinosus* with depleted donor side cytochromes c_2_ (*cycA*) and tetraheme cytochrome subunit (*pufC*), respectively. The gradual oxidation by ferricyanide further reduced the availability of electron donors to *pufC*. Electron transfer through the reaction center was tracked by absorption change and by induction and relaxation of the fluorescence of the bacteriochlorophyll dimer. The rate constants of the electron transfer (~ 3 × 10^3^ s^‒1^) from the mobile donors of *Rvx. gelatinosus* bound either to the RC (*pufC*) or to the tetraheme subunit (wild type) were similar. The electrons transferred through the reaction center dimer were supplied entirely by the donor pool; their number amounted to about 5 in wild type *Rvx. gelatinosus* and decreased to 1 in *pufC* oxidized by ferricyanide. Fluorescence yield was measured as a function of the oxidized fraction of the dimer and its complex shape reveals the contribution of two competing processes: the migration of the excitation energy among the photosynthetic units and the availability of electron donors to the oxidized dimer. The experimental results were simulated and rationalized by a simple kinetic model of the two-electron cycling of the acceptor side combined with aperiodic one-electron redox function of the donor side.

## Introduction

Large protein complexes in bioenergetic membranes are in interaction with small and diffusible redox extrinsic partners to facilitate electron transfer in respiration and photosynthesis [Vasilev et al. 2019; Nawrocki et al. 2019]. The small redox species at or near the membrane surface shuttle electrons between membrane-bound complexes, although their rapid diffusion is confined by the infoldings of the membrane and brought down to the millisecond time range [Lavergne et al. 2009; Vermeglio et al. 2012].

**Fig. 1:**
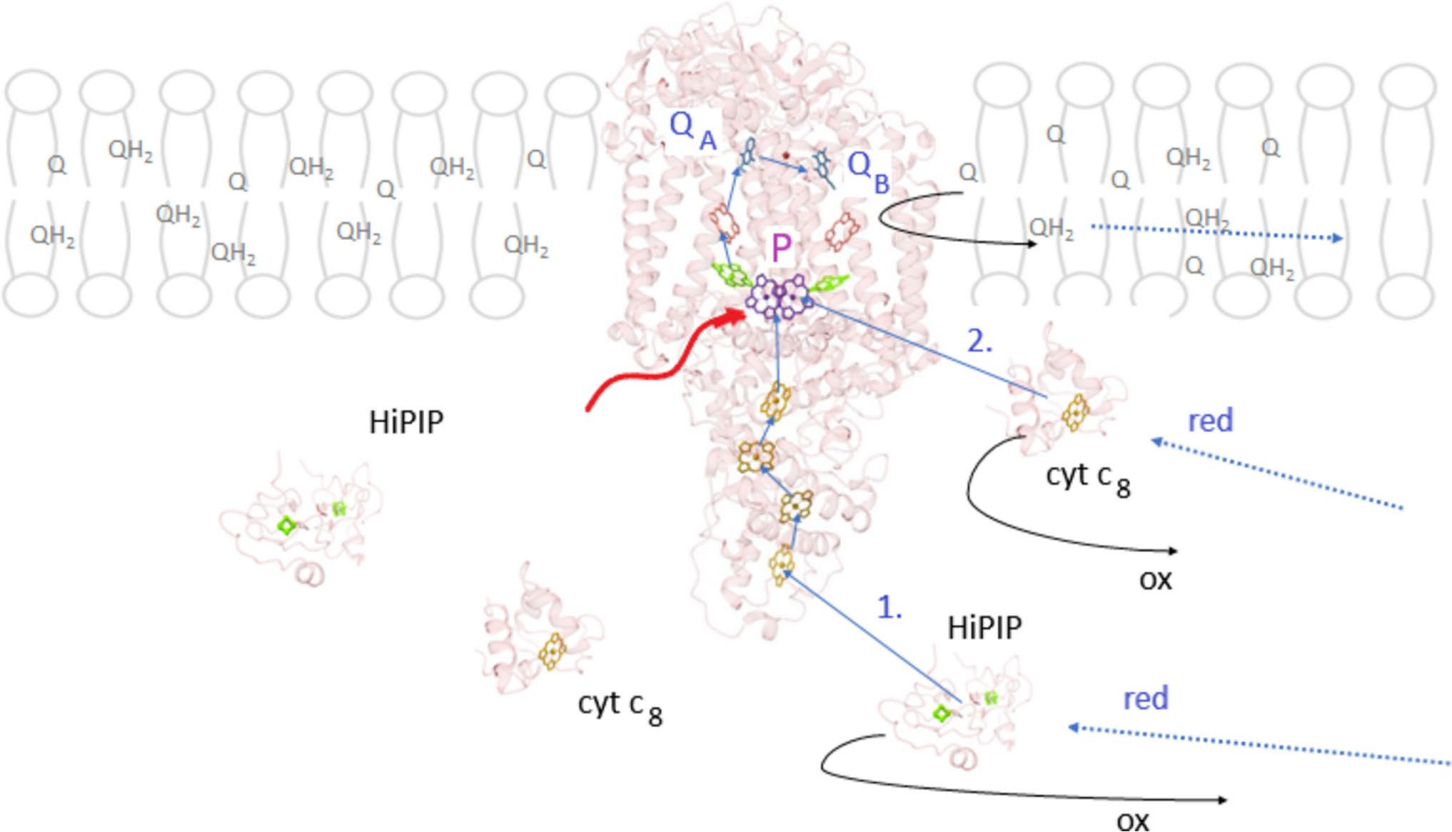
Structure-based demonstration of the initiation of the light-induced cyclic electron flow around the RC. The electron emitted from the excited state of the dimer (P*) upon light excitation (wavy arrow) will run along the A active branch of the cofactors to the secondary quinone Q_B_. The double reduced QH_2_ will be exchanged for Q from the membrane pool and transfer the electrons to the cytochrome *bc*_1_ complex along a time-consuming diffusional pathway (dotted line). On the donor side, the transfer of electrons to P^+^ can occur either from the four hemes of the subunit and is supplied by mobile cytochrome *c*_8_ and/or HiPIP bound to the vicinity of the outmost heme (*Bla. viridis* or *Rvx. gelatinosus*) (route 1) or from cytochrome *c*_8_ and/or HiPIP bound to the RC in the absence of the tetraheme subunit (*pufC* mutant) (route 2). The shuttle of electrons between the RC and *bc*_1_ complexes in the periplasm is also diffusion-controlled (dotted lines). The coordinates of the proteins were taken from the Brookhaven Protein Data Bank with pdb codes of 2jbl (RC), 1isu (HiPIP), and 1a8c (cyt *c*_8_). The figure was drawn using Chimera X software

In bacterial photosynthesis, the electrons are injected from light-excited BChl dimer (P*) and perform cyclic electron transfer between the reaction center (RC) and the cytochrome *bc*_1_ complex via mobile carriers [Mirkovic et al. 2017; Blankenship 2021; Sipka et al. 2022]. The transfer of electrons through the RC is depicted in Fig. 1. The electron carriers on the acceptor side of the RC are quinone molecules located in the hydrophobic membrane region. More diversity characterizes the donor side electron carriers [Gorka et al. 2021]. The RC may (*Blastochloris (Bla.) viridis, Rubrivivax (Rvx.) gelatinosus*) or may not (*Rhodobacter (Rba.) sphaeroides, Rhodobacter (Rba.) capsulatus*) have multiheme subunit with alternating high and low midpoint potentials. The attached tetraheme subunit or the RC itself is donated by periplasmic mobile electron carriers of either soluble cytochromes (c_2_ or c_8_), or membrane anchored c-type cytochrome or high-potential iron–sulfur protein (HiPIP) containing a single cubane [Fe_4_S_4_] cluster [Nagashima et al. 2011]. Cytochrome c_2_ together with its various iso-forms functions in common purple photosynthetic bacteria (*Rba. sphaeroides, Rba. capsulatus*, and *Bla. viridis*). In *Rvx. gelatinosus* cells grown under aerobic conditions in the dark, cytochrome c_8_ is the soluble electron carrier. In contrast, anaerobic conditions in the light evaluate HiPIP as the dominant electron donor. Cytochrome c_8_ and HiPIP bind the RC-bound cytochrome subunit at two distinct but partially overlapping sites.

The interaction between HiPIP and RC (directly or indirectly via tetraheme subunit) is still poorly understood. Except for the case of *Rvx. gelatinosus*, only scarce data are available [Lieutaud et al. 2005]. While, on the one hand, HiPIP is commonly regarded as simple but exotic substitute for the “normal” soluble carrier cytochrome *c*, it may, on the other, have some particularities. Surveys of photosynthetic electron transfer among proteobacterial species show that the participation of HiPIP instead of soluble cytochrome *c* is the rule rather than the exception [Menin et al. 1998]. Therefore, detailed understanding of the interaction of HiPIP with the RC is needed for an exhaustive description of electron donation to the RC in proteobacteria.

The overall stoichiometry of the pools of electron transfer components on the acceptor and donor sides is significantly different. Quinones (mainly ubiquinones but also low-potential quinones as mena- or rhodoquinones) are in large excess to the RC. The membrane sequestered pool can be as large as 20–30 Q/RC [Mascle-Allemand et al. 2008]. All the quinones present in the membrane have an easy access to the Q_B_ binding site. The actual capacity of the pool for reduction is further increased by a factor of two considering the two-electron chemistry of the mobile quinones. On the other hand, the quantity of the soluble periplasmic carriers is generally much (by one order of magnitude) lower in photosynthetically grown bacteria and carry out one-electron chemistry [Lavergne et al. 2009]. If tetraheme subunit is attached to the RC, the electron capacity of the donor pool will increase.

The size of the two-electron pools does not play crucial role under physiological conditions of low cyclic rate. The bottle neck of the steady-state cyclic flow of electrons is the slow confined (not free) diffusion of the mobile carriers over relatively long distances between the complexes. If, however, the intensity of the light excitation is short and large enough to cause multiple turnovers of the RC within the cycling time of the electrons, then the pool of mobile carriers must be alone in fuelling the electrons through the RC as the contribution of the *bc*_1_ complex in the electron transfer will be blocked kinetically. The overall turnover time of the cyclic electron transfer is about 25 ms in vivo under steady-state illumination measured in bacteria *Phaeospirillum molischianum* [Mascle-Allemand et al. 2008]. Although the quinone turnover in the RC (including the two electron and two proton transfer reactions and the Q/QH_2_ exchange) can be fast (about 1.6 ms, [Comayras et al. 2005]), the diffusion and confinements of the reduced quinones delay the shuttle significantly. The kinetics of the donor side reactions can be described similarly: the fast (< 1 ms) oxidation of the electron donor by the RC will be followed by slow diffusion to the *bc*_1_ complex.

Before closing the cyclic flow of electrons around the complexes, several charge separations in the RC can take place upon continuous illumination of high intensity. By tracking the BChl fluorescence induction kinetics, the number of electrons passing through the RC can be derived, although the method and the conditions must be subjects to prior scrutiny. While the subsequent light reactions will close the RC photochemically, the dark inward–outward electron transfers will cause it to re-open. The photochemical closure of the RC works as a dual lock depending on both of Q_A_^‒^ and P^+^ and it is a challenge to separate the contributions of the acceptor and donor sides experimentally [Osváth et al. 1997; Asztalos et al. 2015].

The present work aims to shed new light on the possible pathways of electrons from the donor side to the RC in whole cells of various photosynthetic bacteria with (*Rvx. gelatinosus*) and without (*Rba. sphaeroides*) tetraheme cytochrome subunit of wild type and mutants deficient of attached subunit (*pufC*) or mobile cyt c_2_ pool (cycA). The specific objectives are (1) the comparison of the donation rates of the mobile donors of *Rvx. gelatinosus* bound either to the RC or to the tetraheme subunit, (2) the light-induced competition between the electron pools on the donor and acceptor sides that control the closed/open states of the RC measured by the absorption changes of P/P^+^ and by the induction/relaxation of BChl fluorescence, and (3) the kinetic model of multiple turnovers to describe the electron flow and the redox states of the cofactors. The study will provide deeper understanding of structural, kinetic, and thermodynamic aspects of binding/unbinding of the periplasmic donors to/from the RC.

## Materials and methods

### Bacterial strains and cultivation

Portions of genes encoding periplasmic cytochromes of purple nonsulfur photosynthetic bacteria *Rba. sphaeroides* strain 2.4.1 (*cycA*) and *Rvx. gelatinosus* (*pufC*) were deleted as described earlier [Maróti et al. 2020]. Data of Bioproject accession numbers PRJNA847566 (*Rba. sphaeroides cycA*) and PRJNA847559 (*Rvx. gelatinosus pufC*) are deposited to the Sequence Read Archive (SRA) at the National Center for Biotechnology Information. Bacteria were grown for spectrophotometric analysis in Siström’s medium in filled screw top vessels without oxygen (anaerobic growth) under irradiance of 13 W·m^−2^ from tungsten lamps [Maróti and Wraight 1988]. Under these growth conditions, both cytochromes (c_8_) and HiPIP constituted the periplasmic electron donors in *Rvx. gelatinosus* [Lavergne et al. 2009]. The bacteria were harvested at the exponential phase of the growth at cell concentration of ~ 10^8^ cell·mL^–1^ and were bubbled with nitrogen for 15 min before measurements to preserve the anoxic conditions.

### Chemicals

Myxothiazol and terbutryn were routinely used in 10 μM and 100 μM concentrations, respectively, to inhibit the cyclic electron transfer through the cytochrome *bc*_1_ complex and the Q_A_^‒^ → Q_B_ inter-quinone electron transfer in the RC. To oxidize the periplasmic electron donors of *pufC*, 1 mM potassium ferricyanide was added to the sample [Yoshida et al. 1999; Kis et al. 2022]. Ferrocene was used as external electron donor to P^+^.

### Light-induced P/P^+^ absorption changes

The experiments were carried out by a home-made spectrophotometer [Maróti and Wraight 1988] with some modifications. The absorption changes were induced by high power (2 W) laser diodes (typical wavelength of 808 nm, Roithner LaserTechnik LD808-2-TO3) of variable duration (up to 20 ms). The beam of the measuring light perpendicular to the direction of the excitation came from a stabilized 130 W tungsten lamp, sent through a monochromator (Jobin–Yvon H-20 with concave holographic grating) to select 790 nm wavelength and went through a 1 × 1 cm quartz cuvette that contained the sample. The transmitted measuring light was detected by a photomultiplier (R928 Hamamatsu) protected from the scattered exciting light by a combination of (interference and band pass) optical filters. The detector was connected to a differential amplifier and to a digital oscilloscope (Tektronix TDS 3032). The time resolution of the device was limited to 50 μs. The light-induced signals measured at 790 nm were always related to those at reference wavelengths of 750 nm. To increase the signal-to-noise ratio of the absorption changes, the average of a couple of traces with small repetition rate of (< 0.2 s^−1^) was taken. All measurements were done at room temperature.

As the donor side of *Rvx. gelatinosus* consists of several electron donors (HiPIP and cytochrome *c*_8_) under our growing conditions (anaerobicity in the light), it is recommended to track the oxidation–reduction reactions by detection of the re-reduction of P^+^ rather than the oxidation of the electron donors. While the cytochromes have characteristic intense and sharp absorption band around 550 nm [Kis et al. 2022], the absorption bands of HiPIP are broad and of low intensity around 480 nm [Bartsch 1971]. Consequently, there is no way to detect the oxidation of HiPIP in the presence of highly absorbing cytochromes in intact cells.

### Induction and relaxation of BChl fluorescence

The home-built experimental set-up (BChl fluorometer) and the data processing of fluorescence of intact cells have been described earlier [Kocsis et al. 2010; Maróti et al. 2020]. The fluorescence (900 nm) emitted in the direction perpendicular to the actinic light beam was detected by a near-infrared-sensitive, large-area (diameter 10 mm), and high-gain Si-avalanche photodiode (APD; model 394-70-72-581; Advanced Photonix Inc., USA). A long pass filter (RG 850, Schott) was used to protect the detector from scattered light of the laser and to cut off fluorescence emission from the other pigments than BChl and the base plate. Solutions of extracted BChl or IR-806 dye (Sigma) served as references and were adjusted to the same intensity as that of the fluorescence to avoid the possible artifact coming from the nonlinearity of the response of the detector.

The kinetics of BChl fluorescence (900 nm) was measured during the continuous excitation in induction mode and detected in the dark by testing flashes in relaxation mode. The induction kinetics of fluorescence *F*(*t*) were characterized by the constant part of the instant fluorescence rise (*F*_0_) obtained from interception of the initial data (approximated by straight line) and the vertical axis at *t* = 0, by the maximum level of fluorescence (*F*_max_) and by the normalized variable fluorescence defined as *φ*(*t*) = (*F*(*t*)-*F*_0_)/(*F*_max_-*F*_0_). The fluorescence of the sample during relaxation was probed by a couple (∼ 15) of intense but short (5 μs) laser flashes. The non-exciting character of the testing flashes was checked before each experiment.

### Kinetic simulation

The set of non-linear differential equations describing the kinetics of RC states upon continuous illumination (see Fig. 7) was solved numerically by MathCad 14.0.

## Results

### Kinetic correlation between induction of BChl fluorescence (φ) and P^+^

**Fig. 2:**
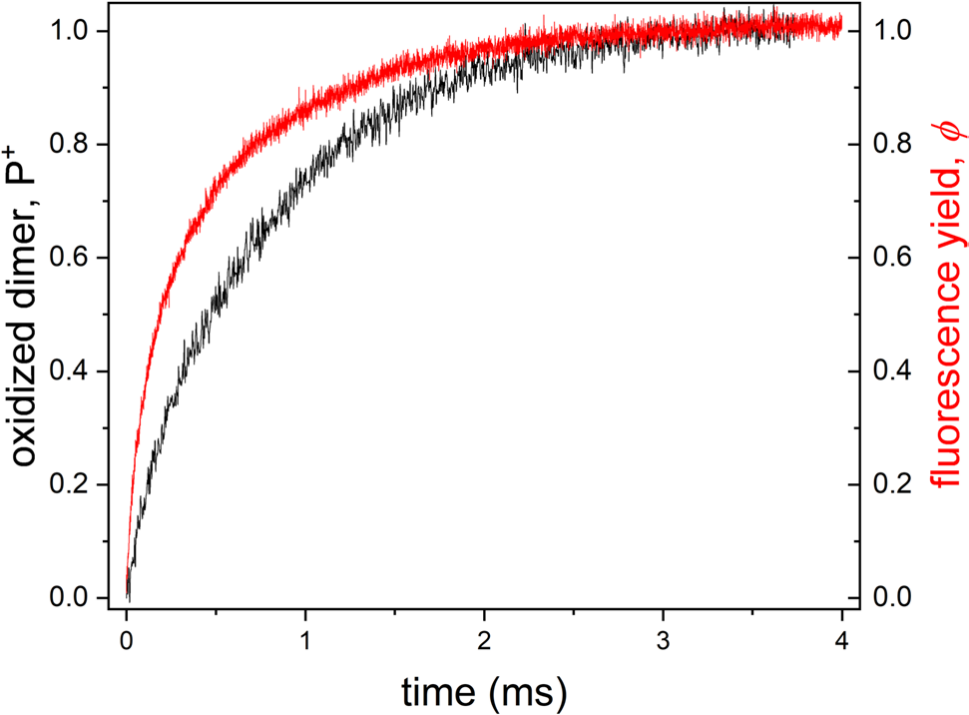
Kinetics of variable fluorescence induction (*φ*) and oxidized dimer (P^+^) of whole cells of *Rvx. gelatinosus* under continuous excitation by a laser diode of wavelength 860 nm and of light intensity 1 W. Both curves were normalized to the saturation values. The BChl fluorescence and the absorption change of P/P^+^ were detected at *λ* > 900 nm and at *λ* = 790 nm vs. 750 nm, respectively

Under continuous light excitation, both the time dependence of the yield of BChl fluorescence and that of oxidized dimer (P^+^) can be directly measured (Fig. 2). The kinetics demonstrate monotonously increasing functions leading to saturations in the (sub)millisecond time range. Depending on the conditions, the RC can be exposed to multiple turnovers. Although the fluorescence induction of the purple bacteria may include multiple phases, it has significantly simpler rise than that of the green plants with so-called “OJIP” elevation [see for recent review Sipka et al. 2021].

The fluorescence induction curve represents the increase of the fraction of photochemically closed RCs during the excitation [Maróti 2016; Sipka et al. 2018]. RC can be closed either by oxidation of the dimer (P^+^), by reduction of the primary quinone (Q_A_^‒^), or by both. The kinetic distinction of the evaluation of the redox states of the RC would assign the time-limiting step of closure of the RC. The kinetics associated with the P^+^ donor (closed) state of the RC is a monotonously increasing function. Unfortunately, the kinetics related to the reduction of the Q_A_ primary acceptor cannot be measured directly. The combined measurement of the fluorescence induction, *φ*(*t*) and the absorption change, P^+^(*t*), however, enables the indirect determination of the kinetics of the closure of the RC at the acceptor side.

The RC can occur in four types of redox states in any time during the excitation:

1 $${\text{PQ}}_{{\text{A}}} + {\text{ P}}^{ + } {\text{Q}}_{{\text{A}}} + {\text{PQ}}_{{\text{A}}}^{ - } + {\text{P}}^{ + } {\text{Q}}_{{\text{A}}}^{ - } = { 1}.$$

The fraction of closed centers is

2 $$x{ } = {\text{ P}}^{ + } {\text{Q}}_{{\text{A}}} + {\text{PQ}}_{{\text{A}}}^{ - } + {\text{P}}^{ + } {\text{Q}}_{{\text{A}}}^{ - } ,$$

which is related to the measured yield of BChl fluorescence via the connectivity parameter between the photosynthetic units *p* [de Rivoyre et al. 2010; Maróti et al. 2020]:

3 $$x = \frac{\varphi }{{1 - p\cdot\left( {1 - \varphi } \right)}}.$$

The amount of P^+^ that can be measured experimentally amounts to

4 $${\text{P}}^{ + } = {\text{ P}}^{ + } {\text{Q}}_{{\text{A}}} + {\text{ P}}^{ + } {\text{Q}}_{{\text{A}}}^{ - } .$$

From these four equations, the kinetics of the redox states PQ_A_^−^ and PQ_A_ can be determined from experimentally measured functions *φ*(*t*) and P^+^(*t*):

5 $${\text{PQ}}_{{\text{A}}}^{ - } \left( t \right) = \frac{\varphi \left( t \right)}{{1 - p \cdot \left( {1 - \varphi \left( t \right)} \right)}} - {\text{P}}^{ + } \left( t \right),$$

6 $${\text{PQ}}_{{\text{A}}} \left( t \right) = \frac{{\left( {1 - p} \right) \cdot \left( {1 - \varphi \left( t \right)} \right)}}{{1 - p \cdot \left( {1 - \varphi \left( t \right)} \right)}}.$$

**Fig. 3:**
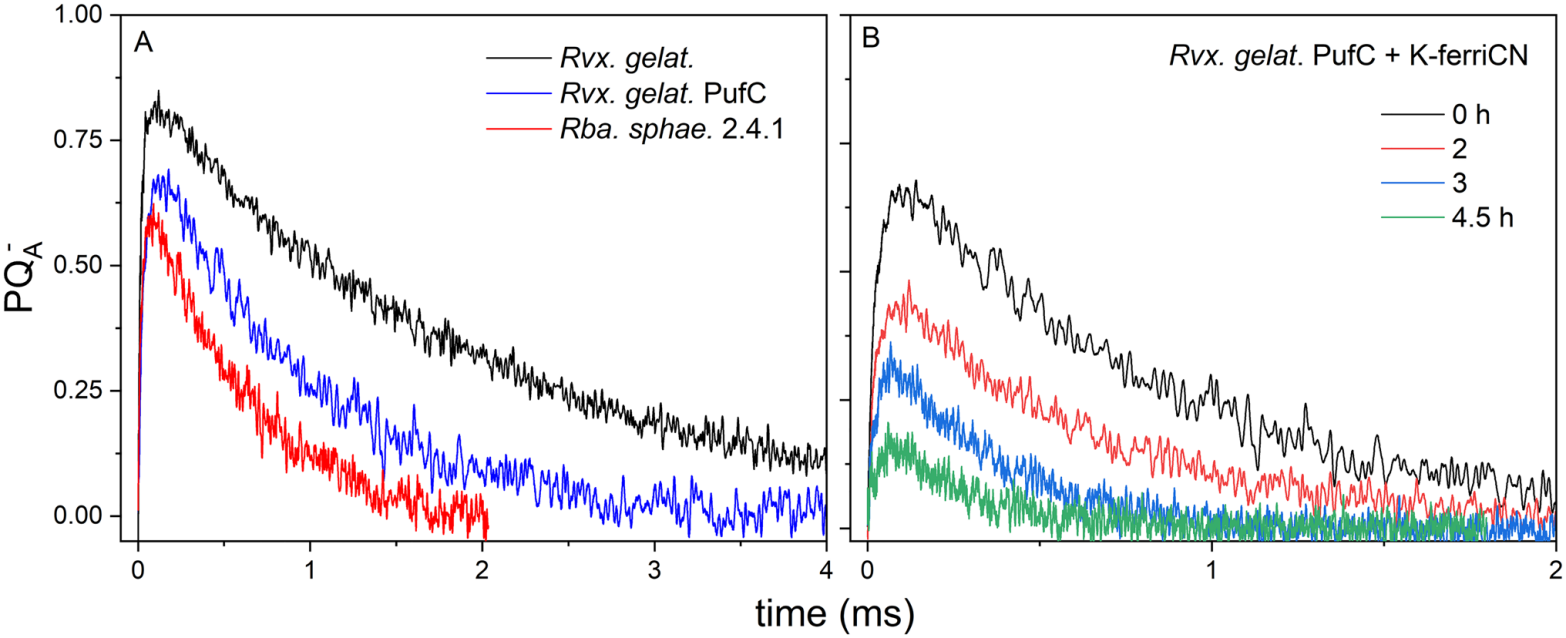
Kinetics of the photochemical closure of the RC on the acceptor side upon continuous excitation: production and disappearance of the reduced primary quinone Q_A_^‒^ in different bacterial strains of *Rvx. gelatinosus*, *pufC* mutant and *Rba. sphaeroides* 2.4.1 (**A**) and in different pool sizes of electron donors in *Rvx. gelatinosus pufC* adjusted by slow oxidation of the donors by 1 mM K-ferricyanide (**B**). The duration of the treatment is indicated

According to Eq. (5), the kinetics of the PQ_A_^–^ state under continuous excitation has a fast initial rise, reaches a maximum, and then begins to decrease monotonously (Fig. 3). The pattern is similar but not identical for bacterial strains of different capacities of electron donation to P^+^. The source of Q_A_^‒^ production is the light-induced charge-separation, and the sink is the first inter-quinone electron transfer. The rate of production of Q_A_^‒^ is proportional to the light intensity and to the rate of P^+^ donation because both increase the rate of charge-separation. The competition of these effects determines the observed Q_A_^–^ kinetics. Therefore, both the location of the maximum and the kinetic parameters are related to the size of the pool of the electron donors. The smaller is the pool, the sooner the maximum occurs, the smaller is the maximum value, and the faster is the decay. The initially produced P^+^ will be re-reduced very quickly by the first (bound) cytochrome donor for all strains. At subsequent turnovers, however, the re-reduction of P^+^ (2.4.1, *pufC* and *Rvx. gelatinous* wild type in that order) will be slower and slower (Fig. 3A). A similar set of kinetics will be obtained if the pool of the electron donors in *Rvx. gelatinosus pufC* is gradually diminished by slow chemical oxidation (Fig. 3B).

The kinetics of the open RC, PQ_A_(t) constructed from Eq. (6) performs monotonously decreasing function with rates dependent also on the capacity and availability of the electron donors (not shown).

### Reopening of the RC after single and multiple turnovers

**Fig. 4:**
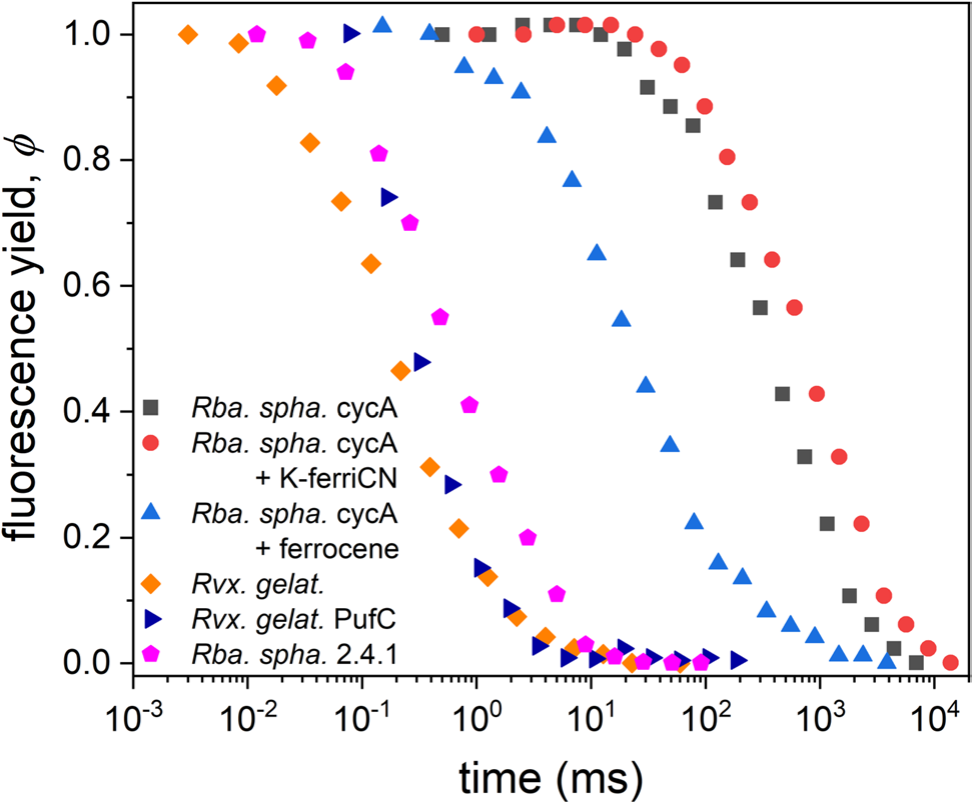
Kinetics of relaxation of the normalized yield of variable BChl fluorescence for strains *Rba. sphaeroides* 2.4.1 (900 μs) and *Rvx. gelatinosus* (300 μs), for their cytochrome deletion mutants *cycA* (500 ms) and *pufC* (400 μs), respectively, and for *cycA* donated by external electron donor ferrocene (35 ms) and oxidized by ferricyanide (1.0 s). The relaxation times are given in brackets. The relaxation was recorded after laser diode excitation (808 nm) of 50 μs duration

While the induction of BChl fluorescence offers information about the closure of the RC, the relaxation describes the kinetics of re-opening of the RC [Asztalos et al. 2015]. It is not surprising that the measured re-opening times of the different bacterial strains vary on a wide range, given that rates on the acceptor and donor sides of the RC could also be so different (Fig. 4). If the time constant of electron donation to P^+^ is much smaller than that of the inter-quinone electron transfer (< 1 ms), then the quinone acceptors will determine the re-opening of the RC (*Rvx. gelatinosus*, *Rba. sphaeroides* 2.4.1 and *pufC*). In the opposite case, where the inter-quinone transfer is faster, or the time constant of donation to P^+^ is larger, the donor side will be the bottleneck of the reopening (*cycA* and its various treatments).

The fast relaxation of the *pufC* mutant of *Rvx. gelatinosus* lacking the tetraheme subunit is somewhat surprising as it is comparable to that of the wild type. This means that the donor binds with high affinity not only to the tetraheme subunit, but also (in the absence of the subunit) to the RC itself, thus the electron transfer rate (at least after the first flash) does not decrease significantly.

### *φ*(P^+^) representation

**Fig. 5:**
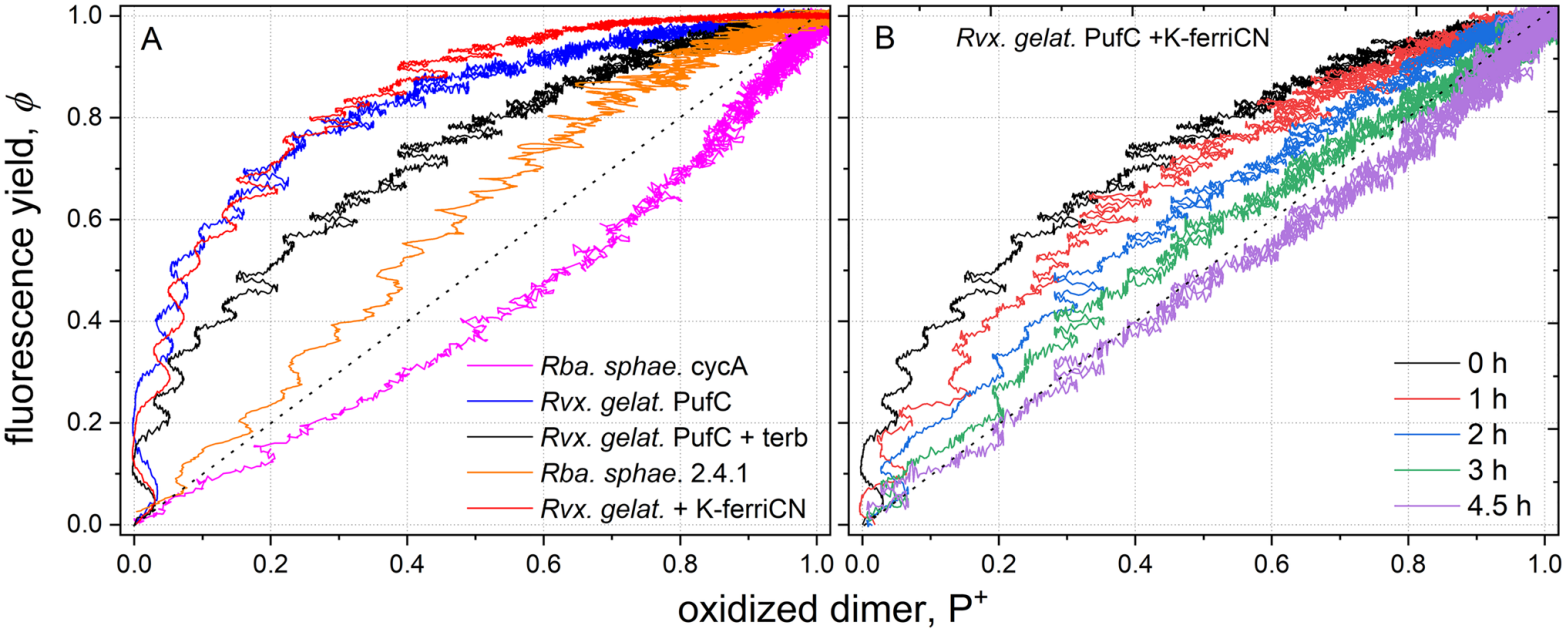
Correlation between the yield of the BChl fluorescence, *φ* and one of its quenchers, P^+^ in bacterial strains of different electron donor systems (**A**) and in *Rvx. gelatinosus pufC* mutant exposed to graduate slow oxidation of the donors by 1 mM potassium ferricyanide (**B**). The duration of the treatment is indicated. The fluorescence yield and the amount of oxidized dimer are normalized to their saturation values

The oxidized dimer is one of the major quenchers of the BChl fluorescence. The measured (normalized) kinetics make it possible to draw direct correlation between *φ* and P^+^ which is a method commonly used to characterize the quenching processes in photosynthesis (Fig. 5). As the kinetics of* φ* and P^+^ are not coinciding (see Fig. 2), the difference can lead to two consequences. (1) If P^+^ can close the RC alone (i.e., Q_A_^‒^ always occurs together with the P^+^ state), then at any time *φ*(*t*) < P^+^(*t*). The difference is due to exciton migration between PSUs, since an exciton hit on a closed RC is not necessarily dissipated in the form of fluorescence but can be utilized photochemically. (2) If P^+^ cannot close the RC alone, then the fluorescence efficiency rises faster than P^+^ does, i.e.,* φ*(*t*) > P^+^(*t*). This suggests that although P^+^ and Q_A_^‒^ are produced together by light excitation, P^+^ is re-reduced by electron donors earlier than the secondary quinone, Q_B_ can re-oxidize the reduced primary quinone, Q_A_^‒^. Such phenomena provide information about the availability of electron donors (Fig. 5A). The larger the capacity of the donor pool (*Rba. sphaeroides* 2.4.1 and *Rvx. gelatinosus*), the more concave the shape of the curve (looking from the bottom). A straight line (bisectrix) is obtained for bacteria with poor (slow) P^+^ donation and with separate PSUs (no energy migration). The curvature will turn to a convex shape if the P^+^ donation remains poor, but the excitons can migrate among the PSUs. This tendency can be tracked in one process if the tetraheme-deficient *Rvx. gelatinosus* mutant, *pufC* is treated by KferriCN, that progressively oxidizes the electron donors and so that no electron donation to P^+^ will occur. In this case, the probability of energy transfer from a closed to an open PSU will determine the convex shape of the curve (Fig. 5B).

### Fluorescence induction: connectivity between PSUs, photochemical rate constant, and number of electrons through the RC

All incoming photons that are not emitted through fluorescence (1‒*φ*) will increase the number of closed RCs (*x*):

7 $$\frac{{{\text{d}}x}}{{{\text{d}}t}} = k_{I} \cdot \left( {1 - \varphi } \right),$$

where *k*_I_ denotes the photochemical rate constant proportional to the light intensity.

According to the exciton diffusion model (lattice mean-field or cluster mean-field approaches, [Maróti et al. 2020]), the supplementary area above the fluorescence induction curve is

8 $${\text{Area}} = \mathop \smallint \limits_{0}^{\infty } \left( {1 - \varphi \left( {t^{\prime}} \right)} \right){\text{d}}t^{\prime} = \frac{1}{{k_{I} }}$$

and the initial slope of the fluorescence induction amounts to

9 $${\text{Slope}} = \frac{{{\text{d}}\varphi }}{{{\text{d}}t}}\left( {t = 0} \right) = \left( {1 - p} \right) \cdot k_{I} .$$

From these equations, the photochemical rate constant (*k*_I_) and the hopping probability of the exciton during the random walk on the network of the RCs (*p*) can be determined by

10 $$k_{I} = \frac{1}{{{\text{Area}}}}\,\,{\text{and}}$$

11 $$p = 1 - {\text{Area}} \cdot {\text{Slope}}$$

Based on these equations, the characteristic values were determined for bacterial strains used in this study (Table 1.)

**Table 1:** Parameters derived from BChl fluorescence induction of *Rba. sphaeroides* 2.4.1 and *Rvx. gelatinosus* wild types and their cytochrome deficient mutants *cycA* and *pufC*, respectively. The standard deviations of the means of the hopping probabilities and photochemical rate constants are indicated

Strain	Initial slope (1/s)	Supplementary area (s)	Hopping probability (*p*)	Photochemical rate constant (*k*_I_, 1/s)
cycA	4.1 × 10^3^	1.9 × 10^–4^	0.22 ± 0.02	(5.2 ± 0.5) × 10^3^
*Rba. sphaeroides* + terb	6.4 × 10^3^	1.3 × 10^–4^	0.20 ± 0.02	(8.0 ± 0.8) × 10^3^
*pufC* + terb	7.6 × 10^3^	1.2 × 10^–4^	0.13 ± 0.02	(8.7 ± 0.9) × 10^3^
*Rvx. gelatinosus* + terb	8.4 × 10^3^	1.1 × 10^–4^	0.10 ± 0.01	(9.3 ± 0.1) × 10^3^

Under our conditions, the *Rba sphaeroides* 2.4.1. strain was characterized by a slightly higher *p* value (0.20–0.22) than that of the *Rvx. gelatinous* strain (0.10–0.13). The small values indicate modest excitonic coupling among the PSUs. Consequently, the initial phase of the fluorescence induction could be described by an exponential rise rather than by a lag phase (see Fig. 2).

**Fig. 6:**
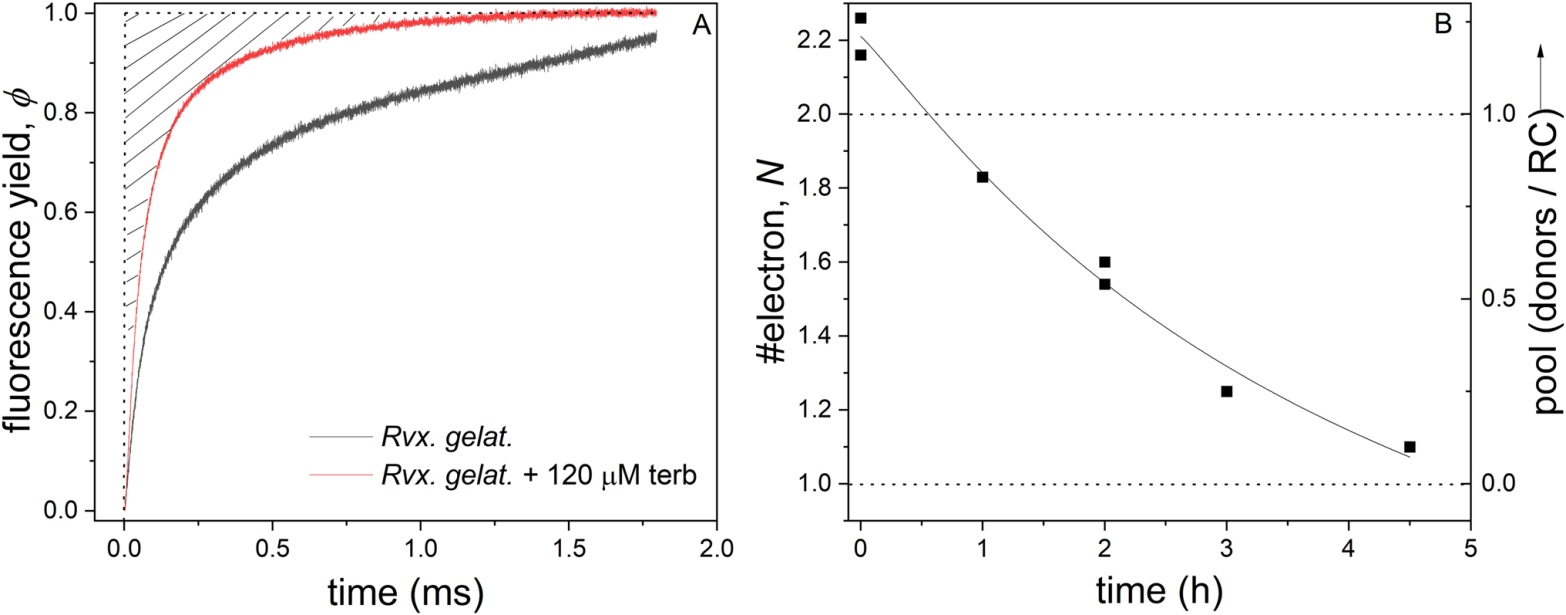
Demonstration (shading) of the supplementary area of the induction of the normalized BChl fluorescence from *Rvx. gelatinosus* blocked by Q_B_ inhibitor terbutryn (**A**). The fluorescence induction of the wild type does not perform saturation on that time scale. The drop of the number of electrons (*N*) passing through the RC and size of the donor pool as a function of duration of the slow oxidation of *pufC* by 1 mM K-ferricyanide (**B**)

The areas above the induction curves (Fig. 6A) are proportional to the number of electrons passing through the RC (see the “performance index” for green plants) [Strasser et al. 2004; Laisk and Oja 2013; Stirbet et al. 2018]. If the supplementary area is related to that of the sample treated with Q_B_ inhibitor terbutryn (where exactly one charge-separation occurs), then the total number of transmitted electrons will be given by.

12 $$N = \frac{{A_{m} \left( {\text{no terbutryne}} \right)}}{{A_{s} \left( {\text{with terbutryne}} \right)}}.$$

Here, *A*_m_ is the full supplementary area up to saturation of *φ*(*t*):

13 $$A_{m} = \mathop \smallint \limits_{0}^{\infty } \left( {1 - \varphi \left( t \right)} \right){\text{d}}t.$$

*A*_m_ is a work integral that measures the energy needed to close all RCs. Subscript “*m*” stands for “multiple,” referring to the multiple turnover leading to the closure of the RCs. The more the electrons from donors to Q_A_ through the RC are transferred, the longer the normalized fluorescence remains lower than 1 and the bigger *A*_m_ becomes. The smallest *A*_m_ denoted by *A*_s_ corresponds to single turnover when every Q_A_ is reduced only once, as in the presence of inter-quinone inhibitor terbutryn.

Particularly, high light intensity and considerable time is needed to saturate the fluorescence of *Rvx. gelatinous* wild type. Consequently, the ratio of the supplementary area measured without and with terbutryn is large, and substantial number of electrons (about 5) are passing through the RC (Table 2.). Smaller numbers characterize the p*ufC* mutant in accordance with the loss of the tetraheme subunit of the RC. Under our conditions, 2.2 charge separations will occur before the pool of the electron donor exhausts. Furthermore, the slow and prolonged oxidation of the pool by ferricyanide results in decreasing number of electrons converging to 1, which value can occur after approximately 4 h of treatment (Fig. 6B).

**Table 2:** The supplementary area of the BChl fluorescence induction (*A*) for multiple (m) and single turnovers (s) and the number of electrons (*N*) in the transport chain in different bacterial strains. The standard deviations of the means of the number of transported electrons are shown

Strain	*A*_m_ (s) (no terb)	*A*_s_ (s) (+ terb)	*N* (= *A*_m_/*A*_s_)
*Rba. sphaeroides* 2.4.1	0.30	0.13	2.37 ± 0.4
*Rvx. gelatinosus*	0.51	0.11	4.76 ± 0.6
*pufC*	0.25	0.12	2.17 ± 0.4

## Discussion

Due to the large intensity of steady-state illumination measured by the high photochemical rate constant, the contribution of the cyt *bc*_1_ complex to the electron transfer was kinetically blocked in the 10–20 ms time range. This was also in agreement with the observation that myxothiazol, a potent inhibitor of the cyt *bc*_1_ complex, did not have any influence on the measured kinetics. The advantages of these simplified conditions can be utilized by delivering direct information about the properties of the electron donors to the RC. The quantitative treatment (simulation) of the multiple turnover of the RC and the possible ways of electron donation of HiPIP to P^+^ in wild type and cytochrome mutants of *Rvx. gelatinosus* will be discussed.

### Simulation of the multiple turnover of the RC

**Fig. 7:**
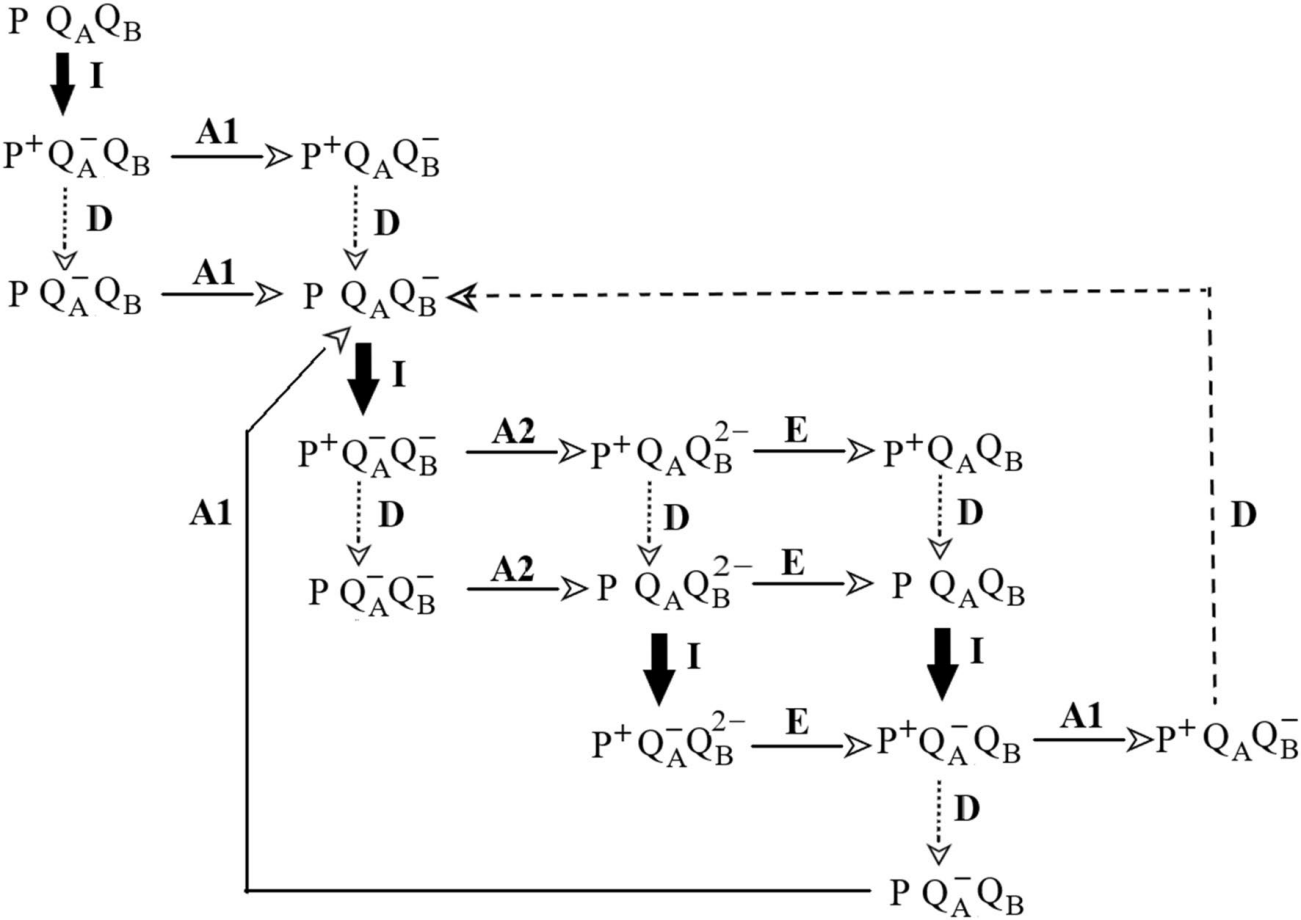
Roadmap of the light-induced multiple electron transfer from periplasmic electron donors to hydroquinone through the RC of *Rvx. gelatinosus pufC* mutant. The cyclic two-electron chemistry of the quinone acceptor side is combined with the acyclic one-electron reactions at the donor side. The light excitation (heavy arrow), the first (A1), and the second (A2) inter-quinone electron transfer and the quinone/quinol exchange (E) are considered as first-order reactions. The donor side reactions (D) of the RC with the pool are approximated by second-order reactions

The donor side electron transfer in *pufC* mutant, where the tetraheme cytochrome subunit attached to RC is missing, is approximated by second-order (collision-based) reaction between P^+^ and a donor from the pool (Fig. 7). The rate constant of re-reduction of P^+^ can be expressed as *D* = *k*_2_·[donor], where *k*_2_ is the bimolecular rate constant limited by the diffusion (*k*_2_ < 1·10^10^ M^‒1^·s^‒1^) and [donor] denotes the concentration of donors in the pool. During successive light reactions upon multiple turnovers, the donor pool together with D decreases progressively. On the acceptor side, the first and second inter-quinone electron transfer and the quinone exchange follow (pseudo) first-order reactions whose rate constants fall within the usual range of 10^3^‒10^4^ s^‒1^.

The simple model is adequate to describe the main features of the kinetic changes of the RC states and of the yield of BChl fluorescence observed in our experiments. Three examples will be provided to show the effectiveness and the power of forecast of the simulation despite the severe simplification of the conditions. Remark: with numeration, follow the original text, please.

1. Calibration of the supplementary area of the fluorescence induction to number of electrons passing through the RC.

The linear relationship between the supplementary area of fluorescence and the number of charges passing through the RC is routinely accepted, although the relationship has not been proven and the conditions of the linearity have not yet been revealed. The model of multiple turnovers outlined above enables us to prove the strict equivalence between the supplementary area and number of electrons.

We must find first the relationship between the normalized yield of fluorescence and the concentration of closed RC using the cluster mean-field approximation [Maróti et al. 2020]:

14 $$\varphi = \left( {1 - p} \right) \cdot x + \left( {1 - p} \right) \cdot p \cdot x^{2} + p^{2} \cdot \left( {\frac{{x^{2} }}{4} + \frac{{3 \cdot x^{3} }}{4}} \right).$$

The donor pool provides multiple turnovers of the RC. The larger the pool, the more turnovers can occur, and the more charges (electrons) can pass through the RC. From the pool size specified during the simulation, we can determine the number of charges passing through RC and the kinetics of the fluorescence induction. The calculated supplementary area of fluorescence induction increases linearly to the calculated number of electrons passing through the RC (data not shown). If the supplementary area is normalized by the rate constant of the photoexcitation *k*_I_, it will offer the number of electrons directly. The linear relationship is independent on the value of the hopping parameter *p*. The increase of the area is due to the deceleration of the fluorescence kinetics compared to a single (fast) turnover. The rise of the fluorescence shifts toward the larger time domain, thereby increasing the supplementary area (see Fig. 6A).

**Fig. 8:**
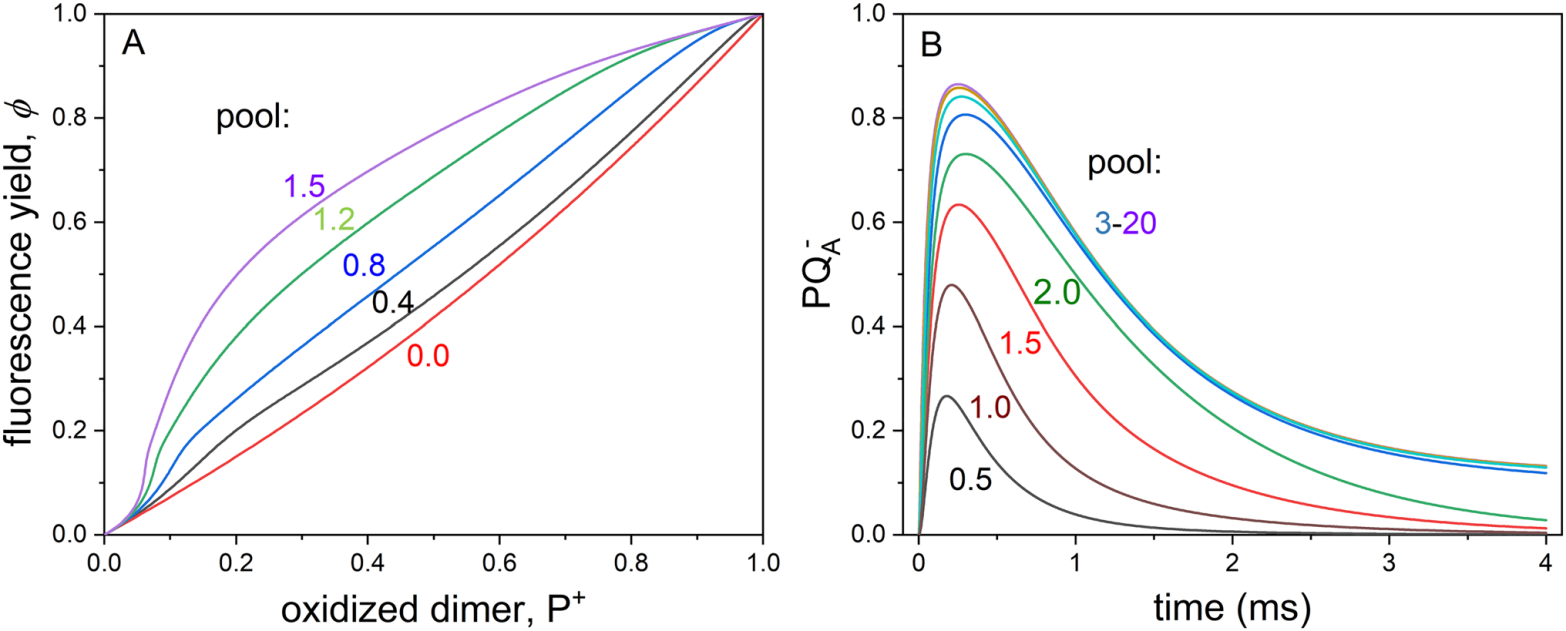
The effects of the pool size of the electron donor on stoichiometry and kinetics simulated by numerical calculations based on Fig. 7. Panel A: relationship between normalized yield of BChl fluorescence *φ* and normalized concentration of oxidized dimer P^+^ after variable duration of treatment of the *pufC* mutant by ferricyanide. The following parameters with Eq. (14) were used: *k*_I_ = 8 × 10^3^ s^−1^, *k*_2_ = 1 × 10^10^ M^−1^·s^−1^, [RC] = 8 μM, *A*_1_ = 3 × 10^4^ s^−1^, *A*_2_ = 2 × 10^3^ s^−1^, *E* = 1 × 10^2^ s^−1^ and *p* = 0.3. The size of the donor pool changes from 0 to 1.5 donor/RC. Panel B: rise and decay of the PQ_A_^‒^ redox state of the RC simulated with the parameters of *k*_I_ = 2·10^4^ s^−1^, *k*_2_ = 6·10^9^ M^−1^ s^−1^, [RC] = 3 μM, *A*1 = 3 × 10^3^ s^−1^, *A*2 = 1 × 10^3^ s^−1^ *E* = 1 × 10^2^ s^−1^ and *p* = 0.3. The size of the pool was given in units of donor/RC

- 2) The simple model can explain the observed change of *φ* vs. P^+^ plot from concave to convex curvature upon decrease of the size of the donor pool if the tetraheme-deficient *Rvx. gelatinosus* mutant *pufC* is treated by ferricyanide (Fig. 8A). The slow and progressive oxidation of the mobile periplasmic electron donors is accompanied by transition from concave to convex shape of the curve observed in our experiments (Fig. 5B). The comparison with the experimental results is satisfactory. Although the change is modest, it is very sensitive to the size of the pool: the transition performs between 0 and 1.5 donor/RC under our numeric values. Additionally, the simulation can offer strong support of the larger scale of variations of the *φ* vs. P^+^ plots measured for different strains (Fig. 5A). These numerical simulations can quantify the capacity of the electron donors in these bacteria (not shown).

- 3) The simulated set of kinetics of the PQ_A_^‒^ redox state of the RC with variable sizes of the pool of the electron donors upon continuous illumination is depicted in Fig. 8B. The comparison with the actual set of measurements (Fig. 3) is acceptable: the kinetics and amplitudes of the corresponding phases of the rise and the decay and their dependence on the pool size show reasonable agreement. Similar correlation with the measured kinetics can be obtained also for the kinetics of the open RC (not shown). Despite the omission of the specific details of the constraint of the diffusion and of the kinetic consequences of the exhaustion of the pool of the electron carriers, the simulations based on our model (Fig. 7) fit nicely to all our absorption change and fluorescence measurements.

### Direct and indirect (via tetraheme subunit) electron donation from HiPIP to the RC

The electron transfer from HiPIP and to the tetraheme subunit proceeds with a *t*_1/2_ = 300 μs determined in whole cells for *Rvx. gelatinosus* [Schoepp et al. 1995]. A similar value was obtained from our fluorescence relaxation experiment (Fig. 4). As the HiPIP forms a complex with the cytochrome subunit of the RC in *Rvx. gelatinosus*, the large time constant can therefore not be explained by a diffusion-controlled process but must be rationalized by taking account of the docking site and of the free energy of the reaction. Marcus's theory of electron transfer, together with the empirical determination of the relevant parameters, allows calculation of the electron transfer rate as a function of the edge-to-edge distance between the two cofactors, the free energy difference, and the reorganization energy of the reaction [Moser et al. 1992]. Solving the Marcus equation by using the relevant values of the redox midpoint potentials and by adopting the docking geometry, Alric et al. (2004) were able to reproduce the experimentally determined t_1/2_ of 300 μs in whole cells of the *Rvx. gelatinosus*.

More details were obtained from co-crystallization of the two proteins from *Tch. tepidum* [Kawakami et al. 2021]. The structure suggests that HiPIP interacts with the outermost heme of the subunit (heme 1) through its hydrophobic surface and with the second low-potential heme. The binding interface between the HiPIP and the cytochrome subunit is primarily comprising of uncharged residues and the interaction is primarily hydrophobic. Due to the thermodynamically unfavorable interprotein electron tunneling from HiPIP (*E*_m_ = 340 mV) to heme 1 (*E*_m_ < 150 mV), this electron transfer is the rate-limiting step in the entire pathway of electron to the oxidized special pair in the RC.

However, the electron transfer will become thermodynamically favorable if the HiPIP or cyt c_8_ binds directly to the docking site of the RC. The high midpoint redox potential of P/P^+^ (~ 500 mV) assures downhill electron transfer from HiPIP (or cyt c_8_) to P^+^. The favorable free energy gap can compensate the docking which is probably not optimal to electron transfer in the Marcus expression of the rate calculation. This agrees with the results of the fluorescence relaxation kinetics where the re-opening of the closed RC remained fast in the *pufC* mutant (~ 400 μs) and was close to that of the wild type (~ 300 μs). Although the binding of HiPIPs to the RC is not a physiologically determined process, it is working close to that of the WT. This may be attributed to the dominance of the hydrophobic interactions of the docking that enables closer binding than electrostatics would allow as is the case for cytochrome *c*_2_ [Alric et al. 2004; Axelrod et al. 2009; Kawakami et al. 2021]. HiPIP is strongly associated not only to the RC but to other membrane-integral reaction partners, as well. The tight complex hinders rapid exchange of a photo-oxidized HiPIP for another reduced HiPIP (product inhibited reaction) which may limit the rate of turnover. A similar effect was described for cytochrome c in isolated RC of *Rba. sphaeroides* [Gerencsér et al. 1999]. Additionally, HiPIP has a strong tendency to multimerize in solution [Lieutaud 2004]. The high local concentration of HiPIP in the periplasm will decrease the relative portion of the monomers and therefore its binding to the RC.

The HiPIPs are usually considered as straightforward alternatives to the soluble cytochromes just like the small copper proteins in some proteobacteria or in oxygenic photosynthesis. However, the exceptional features of the HiPIPs deduced from these studies reveal that they are not simple substitutes for soluble cytochromes in proteobacteria. The HiPIPs can satisfy the unique demands of these interactions between protein complexes and periplasmic mobile electron donors in photosynthetic bacteria.

## Conclusions

The binding of the electron donor to the RC, the electron transfer from donor to RC, and the persistence of the post-electron transfer state (unbinding) last for a couple of milliseconds and are compatible with rates of cyclic photosynthetic electron transfer at physiologically relevant light intensities. Kinetic constraint of the fast turnover of the RC will be evoked upon intense photoexcitation and reduced capacity of electron donors in bacterial strains with deleted cytochromes (*cycA* and *pufC*). It has been shown that under these conditions, the rate of electron transfer will be limited by the availability of the periplasmic electron donors. The results provide new insight into the critical step of the long-range trans-protein electron transfer in photosynthetic organisms.

## Data Availability

All data and material are available from the corresponding author.

## Abbreviations

BChl: Bacteriochlorophyll
Cyt: Cytochrome
*F*: BChl fluorescence
*φ*: Normalized yield of the variable fluorescence
HiPIP: High potential iron–sulfur protein
*N*: Number of electrons through the RC
*p*: Probability of hopping of the excitons
P: BChl dimer of the RC
PSU: Photosynthetic unit
Q_A_, Q_B_: Primary and secondary quinone acceptors, respectively
RC: Reaction center
